# *Malagasyprinus*, a new genus of the Saprininae from Madagascar with description of two new species (Coleoptera, Histeridae, Saprininae) (First contribution to the knowledge of the Histeridae of Madagascar)

**DOI:** 10.3897/zookeys.333.5909

**Published:** 2013-09-20

**Authors:** Tomáš Lackner, Yves Gomy

**Affiliations:** 1 Czech University of Life Sciences, Faculty of Forestry and Wood Sciences, Department of Forest Protection and Entomology, Kamýcká 1176, CZ-165 21 Praha 6 – Suchdol, Czech Republic; 2 2 Boulevard Victor Hugo, 58000 Nevers, France

**Keywords:** *Malagasyprinus*, Histeridae, Saprininae, taxonomy, Madagascar

## Abstract

Based on the results of recent phylogenetic analysis of the higher taxa of the Saprininae as well as external morphological characters, especially the presence of deep and large prosternal foveae, and the shape and position of the sensory organs of the antennal club, the species *Saprinus* (s.str.) *caeruleatus* Lewis, 1905 is excluded from the genus *Saprinus* and a new genus *Malagasyprinus*, exclusive to Madagascar, is established for it. The new genus shows mainly characters that are apomorphic for the subfamily and contains another two, highly similar allopatric species *Malagasyprinus perrieri* **sp. n.**, and *Malagasyprinus diana* **sp. n.**, described herein. The three species are best separated from each other by the structure of the prosternum and male terminalia, especially the shape of the aedeagus. We re-describe *Malagasyprinus caeruleatus* **comb. n.** and provide *Malagasyprinus perrieri* and *Malagasyprinus diana* with brief differential diagnoses. All taxon descriptions are accompanied with color habitat photographs, SEM micrographs and drawings of their male genitalia. A key to the species of *Malagasyprinus* is given. Sensory structures of the antenna of *Malagasyprinus caeruleatus* **comb. n.** are likewise depicted herein. The systematic position of the newly erected genus is discussed. A lectotype of *Saprinus caeruleatus* Lewis, 1905 is designated.

## Abstract (fr)

Se référant aux résultats des récentes analyses phylogénétiques portant sur les taxa supérieurs des Saprininae ainsi que sur des caractères morphologiques externes comme les larges et profondes fovéoles prosternales et les forme et position des organes sensoriels des massues antennaires, l’espèce *Saprinus* (s.str.) *caeruleatus* Lewis, 1905 est exclue du genre *Saprinus* et un nouveau genre *Malagasyprinus*, endémique de Madagascar, est établi pour le recevoir. Le nouveau genre présente principalement des caractères apomorphes pour les Saprininae, et contient aussi deux autres espèces allopatriques et similaires *Malagasyprinus perrieri* **sp. n.** et *Malagasyprinus diana* **sp. n.** décrites ici. Les trois espèces sont bien séparées les unes des autres par la structure de leurs prosternums et par leurs genitalia mâles, spécialement la forme des édéages. Nous re-décrivons *Malagasyprinus caeruleatus* **comb. n.** et donnons de courtes diagnoses différentielles de *Malagasyprinus perrieri* et *Malagasyprinus diana*. Toutes les descriptions sont accompagnées des photographies d’habitus en couleurs, de photographies au microscope à balayage (MEB) et de dessins des genitalia mâles. Un tableau des espèces de *Malagasyprinus* est donné. Les structures sensorielles antennaires de *Malagasyprinus caeruleatus* sont également représentées. La position systématique du nouveau genre est discutée. Le lectotype de *Saprinus caeruleatus* Lewis, 1905 est désigné.

## Introduction

Lewis (1905) described the species *Saprinus caeruleatus* based on a single specimen originating from southern Madagascar: Plateau de l’Androy, Région d’Ambovombe, adding a significant and explicative remark: ‘I do not know of any species similar to this’. This assessment was perhaps why Dahlgren (1969: 263, fig. 2:A; 267, fig. 3:I) depicted drawings of apices of the 8^th^ abdominal sternite of male as well as aedeagus based on the examination of four male specimens he found at MNHN collected by H. Perrier de la Bâthiein 1906. Probably because Dahlgren (1969) believed that he was dealing with such a well-characterized species he did not bother with the examination of its type specimen, housed at NHM. In this fashion, the identification process of specimens of ‘*Saprinus caeruleatus*’ has been simple and quick among the specialists on the Histeridae.

While mounting a larger series of this ‘species’ based on old as well as new collections, the junior author noticed significant differences between the aedeagi among the specimens. In the light of this discovery, the minor differences in external morphology, hitherto attributed to individual variation, suddenly gained significance. Furthermore, the senior author, when entrusted with a lot of recently collected specimens of ‘*Saprinus caeruleatus*’ discovered among them yet another, highly similar species. Based on these examinations, it became evident that ‘*Saprinus caeruleatus*’ is, in fact, a complex of three sibling species, which are described and compared below. Fortunately, the sole type specimen of *Saprinus caeruleatus*, housed in NHM is a male and allowed us to fix its identity in the light of the discovery of the other new species, based on the male genitalia (especially aedeagus). The three species, although very similar externally, differ in the male terminalia as well as in external characters, and are also allopatric: while *Saprinus caeruleatus* has so far been collected only in the southern part of the island, one of the newly described species occurs only in the west to north-west of Madagascar, while the other newly described species is found only in the northernmost tip of the great island (Fig. 62). As to Dahlgren’s (Fig. 25) depictions of the eighth sternite and the apex of aedeagus, his drawings must be attributed to one of the newly described species, since the specimens he examined originated from Maevatanana (*former* Suberbieville), which is in the north-west of Madagascar.

During the PhD studies of the senior author a large number of species belonging to the Saprininae subfamily has been examined. Upon closer examination of *Saprinus caeruleatus*, marked differences from the rest of the members of the genus were noticed and therefore a decision to include this species in the phylogenetic analysis of the subfamily was taken. Based on the results of the analysis (Lackner, unpublished), together with careful examination of the external morphological characters we decided to erect a new genus for the above-mentioned taxon that is characterized below.

## Material and methods

All dry-mounted specimens were relaxed in warm water for several hours or overnight, depending on the body size. After removal from original cards, the beetles were side-mounted on triangular points and observed under a Nikon 102 stereoscopic microscope with diffused light. Some structures were studied using methods described by Ôhara (1994): the antenna and male genitalia were macerated in a hot 10% KOH solution for about 15 minutes, cleared in 80% alcohol, macerated in lactic acid with fuchsine, incubated at 60°C for two hours, and subsequently transferred into a 1:1 mixture of glacial acetic acid and methyl salicylate heated at 60°C for 15 minutes and cleared in xylene. Specimens were then observed in α-terpineol in a small glass dish. Digital photographs of the male terminalia and antenna were taken by a Nikon 4500 Coolpix camera and edited in Adobe Photoshop CS4. Based on the photographs or direct observations, the genitalia and antennal structures were drawn using a light-box Hakuba klv-7000. SEM micrographs of *Saprinus caeruleatus* were taken with a JSM 6301F microscope at the laboratory of Faculty of Agriculture, Hokkaido University, Sapporo, Japan; while SEM micrographs of the newly-described species *Malagasyprinus perrieri* sp. n. and *Malagasyprinus diana* sp. n. were taken at the Laboratory of the Electron Microscopy at the Faculty of Biology, Charles University, Prague, Czech Republic. Habitat photographs of *Malagasyprinus perrieri* sp. n. were made by G. Goergen (Cotonou, Benin) and those of *Malagasyprinus caeruleatus* comb. n. and *Malagasyprinus diana* sp. n. were made by F. Slamka (Bratislava, Slovakia). All available specimens were measured with an ocular micrometer. Beetle terminology follows that of Ôhara (1994) and Lackner (2010). Separate lines of the same label are marked by slash (/). The following acronyms of museums and private collections are used throughout the text:

CAS California Academy of Sciences, San Francisco, USA (D. Kavanaugh);

CYG Yves Gomy collection, Nevers, France;

MNHN Muséum National d’Histoire Naturelle, Paris, France (A. Taghavian);

NHM The Natural History Museum, London, United Kingdom (R. Booth);

TLAN Tomáš Lackner collection, temporarily housed at NCB Naturalis, Leiden, Netherlands.

### Abbreviations used in measurements

PEL Length between anterior angles of pronotum and apices of elytra;

APW Width between anterior angles of pronotum;

PPW Width between posterior angles of pronotum;

EL Length of elytron along sutural line;

EW Maximal width between outer margins of elytra.

## Taxonomy

### Malagasyprinus gen. n.

http://zoobank.org/E01181D5-08DB-4651-98F7-7DFFE6106BB2

http://species-id.net/wiki/Malagasyprinus

#### Type species.

*Saprinus caeruleatus* Lewis, 1905.

#### Diagnosis.

Rather small Saprininae histerid (PEL 2.05–2.60 mm) with black body, brown to black elytra; dorsally with blue metallic tinge; legs and antennae paler than the rest. Frons rugulose-lacunose, coarsely and densely punctured, depressed; frontal stria widely interrupted anteriorly, prolonged onto clypeus, sometimes difficult to discern and appearing complete; sensory structures of antenna in form of a single sensory area with a corresponding stipe-shaped vesicle situated on internal distal side of the antennal club (Fig. 22); eyes large and strongly convex; pronotal hypomeron asetose; pronotal foveae (sensu Lackner 2010: 38, fig. 146) absent, pronotum with variously deep longitudinal lateral depression separated from the pronotal margin by a slightly convex punctate band, median part of pronotum moderately to strongly convex, entire pronotal disc with coarse punctures, lateral longitudinal depression and surface around it with extremely rugose and deep longitudinal wrinkles; marginal pronotal stria carinate laterally, slightly bi-sinuate; entire elytral disc (with exception of small, occasionally punctate ‘mirror’ on fourth elytral interval) coarsely verrucose-punctate; dorsal elytral striae obliterated by punctuation, represented occasionally only by their basal fragments; basal fragment of fourth dorsal elytral stria and basal third to half of sutural elytral stria present as a rule, connected; humeral elytral stria usually discernible. Prosternal foveae (pre-apical foveae of Lackner 2010: 41, fig. 148) large and deep; both sets of prosternal striae present; prosternal process in two species depressed on anterior two-thirds; underside of the body with variously coarse and dense punctuation (depending on species).

#### Differential diagnosis.

Externally this new taxon at first glance resembles a specimen of the genus *Saprinus* s. str. (the type species of this genus has originally been a *Saprinus*), but the shape of the sensory structures of the antenna should distinguish it from *Saprinus* immediately (compare Figs 22 and 23). Furthermore, the deep longitudinal pronotal wrinkled depression with convex median part of the pronotum, and large and deep prosternal foveae quickly separate it from the members of *Saprinus* as well. However, prosternal foveae are present among the members of the primarily Palaearctic subgenus *Hemisaprinus* Kryzhanovskij in Kryzhanovskij and Reichardt 1976 of the genus *Saprinus* Erichson, 1834 but they are never as deep and large as in this newly erected genus, and, furthermore, the pronotal depressions (pronotal foveae of Lackner 2010: 38, fig. 146) are present in *Hemisaprinus*, whereas they are absent in *Malagasyprinus*. The most marked differences between *Hemisaprinus* and *Malagasyprinus* are found in the structure of their sensory areas of the antenna: the sensory structures of the antennal club of the type specimen of the subgenus *Hemisaprinus*, *Saprinus (Hemisaprinus) subvirescens* (Ménétriés, 1832) are similar to those of *Saprinus semistriatus*, and consist of four ovoid sensory areas on ventral side and one vesicle situated under internal distal margin (compare Figs 22 and 24). In order to distinguish this newly erected genus from other Afrotropical genera, the reader is referred to the key by the senior author (Lackner 2013: 66). Although this key features only the species “*Saprinus caeruleatus*”, it is well applicable for all members of *Malagasyprinus*.

#### Biology.

Series of *Malagasyprinus caeruleatus* comb. n. have been collected in a dry forest by a pitfall trap baited with fish. Specimens of *Malagasyprinus perrieri* sp. n. and *Malagasyprinus diana* sp. n. have been collected by beating the bushes, as well as by pitfall traps.

#### Distribution.

Madagascar.

#### Etymology.

The name of this newly erected taxon is a combination of the genus name *Saprinus* with a prefix derived from the epithet suggesting Madagascar origin. Gender masculine.

### Malagasyprinus caeruleatus

(Lewis, 1905) comb. n.

http://species-id.net/wiki/Malagasyprinus_caeruleatus

Figs 1 –22

Saprinus caeruleatus Lewis, 1905: 611; Mazur (1997): 220; Mazur (2011): 180.

#### Type locality.

Madagascar, Androy region, Ambovombe.

#### Type material examined.

**MADAGASCAR:** LECTOTYPE (present designation): Male, mounted in Entofix on its right side, with male terminalia extracted and glued to the same card, with the following labels: “♂”; (white, hand-written label); “Plateau de l’Androy, rég. D’Ambovombe” (light-blue, printed label); “Madagascar” (white label, narrow and long, printed); “G. Lewis Coll. B.M. 1926-369” (white label, elongate, printed and characteristic of the specimens originating from G. Lewis’ collection); “*Saprinus caeruleatus* Lewis Type” (white, hand-written label of Lewis); “TYPE” (white round label with red margin, printed); “Y. Gomy des. Lectotype” (red, printed label); “*Saprinus* (s.str.) *coeruleatus* Lew., Y. Gomy Det. 2006” (printed-written determination label) (NHM).

#### Additional material examined.

5 ♂♂ & 3 ♀♀ and 49 exs. (sex undetermined) “Mikea Forest / Feb. 2004”; dry forest / fish baited trap / Ilkka Hanski leg”; 1 ♀: “Toliara / Prov., Ranobe, elev. 30m / 23°02'03"S, 043°36'43"E / 5–9 February 2003; Frontier Wilderness / Project, sifted litter (leaf mold / rotten wood) in spiny forest / thicket code: MGF056”; 1 ♀, “Madagascar Sud-Ouest / LAMBOMAKANDRO 500m / Tuléar / vii-57 Andria R”; “Institut / Scientifique / MADAGASCAR”.

**Re-description.** Body measurements: PEL: 2.05–2.30 mm; APW: 0.75–0.85 mm; PPW: 1.65–1.80 mm; EL: 1.10–1.30 mm; EW: 1.90–2.10 mm.

**Figures 1–2.**
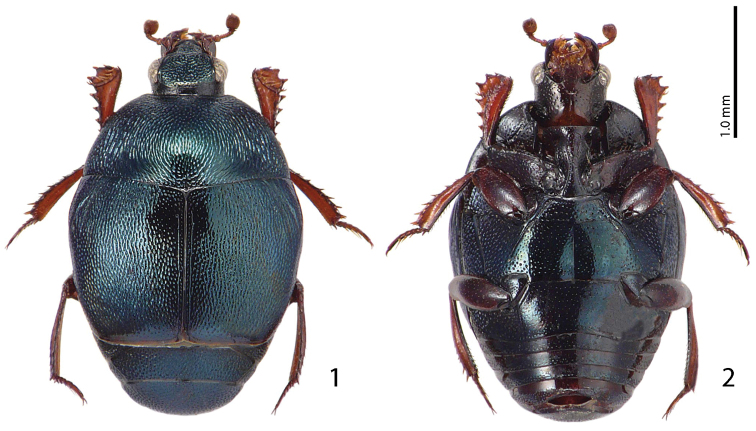
*Malagasyprinus caeruleatus* (Lewis, 1905) comb. n. **1** habitus dorsal view **2** ditto, ventral view.

**Figures 3–11.**
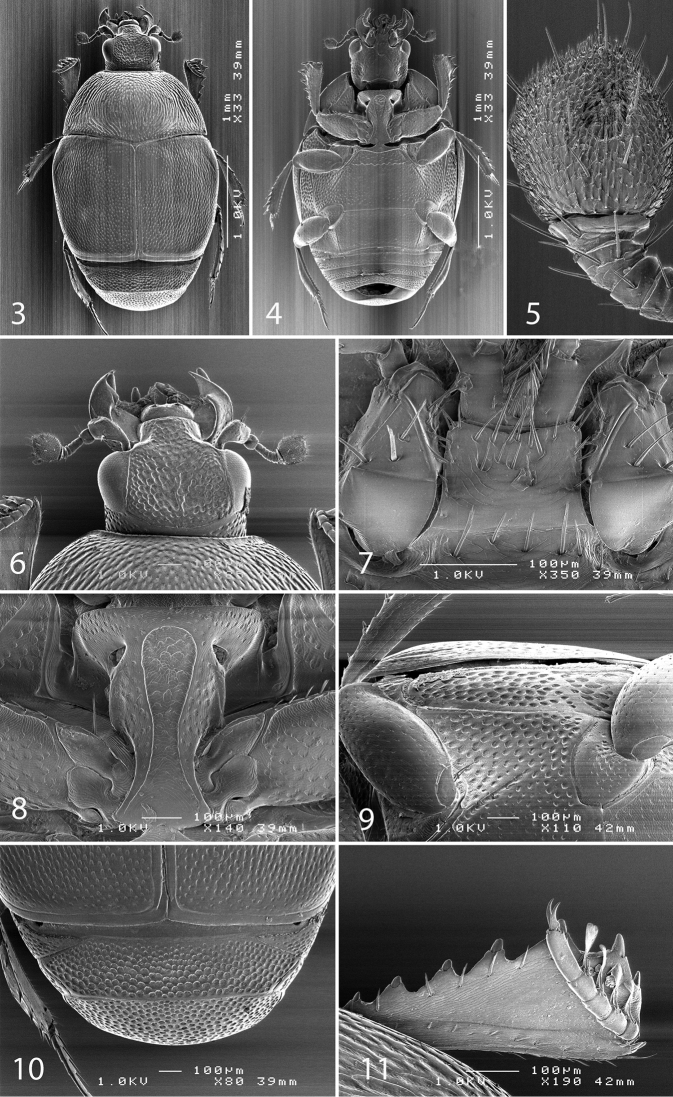
SEM micrographs *Malagasyprinus caeruleatus* (Lewis, 1905) comb. n. **3** habitus dorsal view **4** ditto, ventral view **5** antennal club, ventral view **6** head, dorsal view **7** mentum, ventral view **8** prosternum **9** lateral disk of metaventrite and metepisternum **10** propygydium and pygidium **11** protibia, dorsal view.

Body (Figs 1–4) roundly oval, convex, cuticle entirely pitch-black with dark blue metallic hue, shining; legs, mouthparts and antennae light red-brown.

**Figures 12–21.**
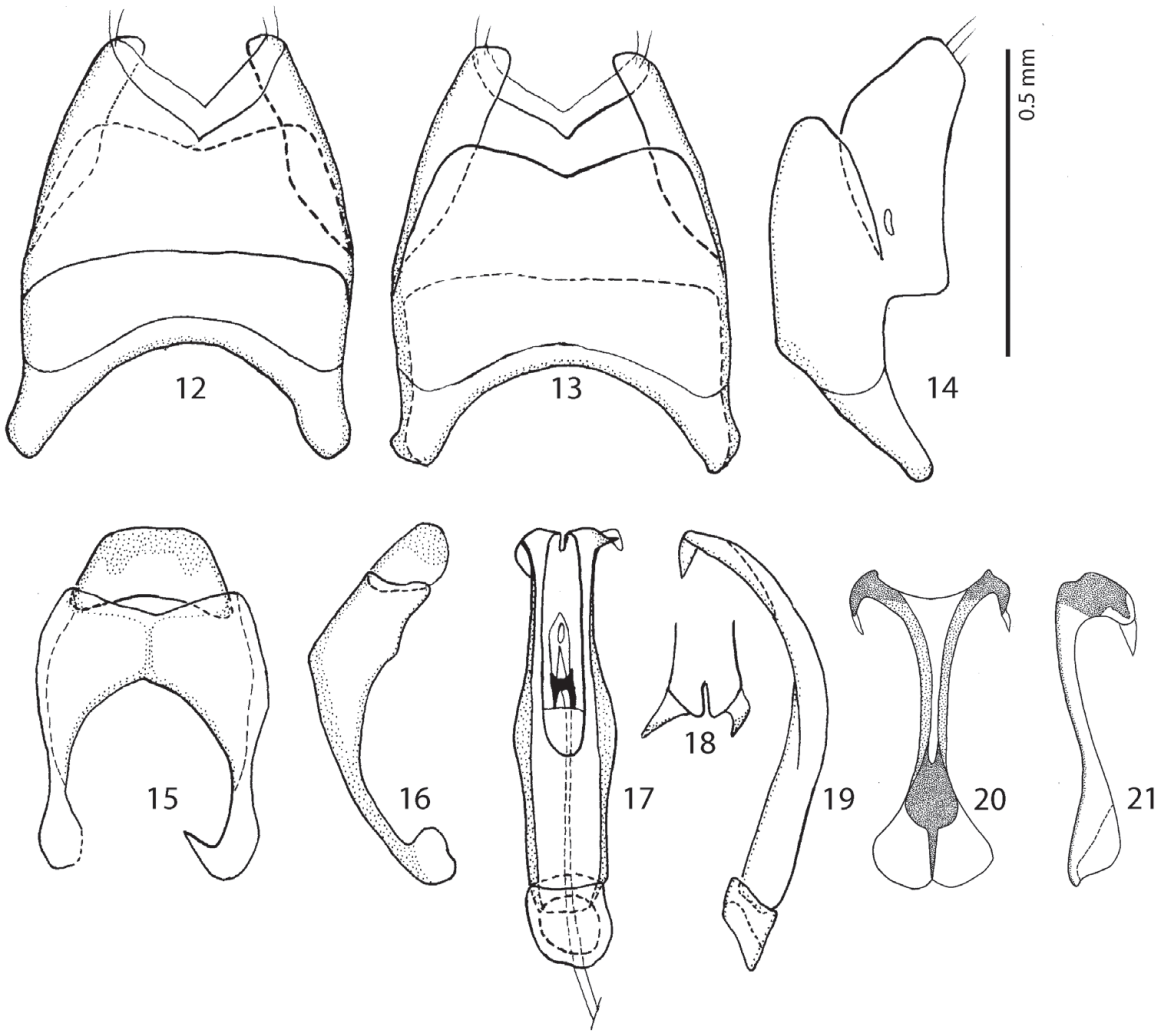
Male terminalia *Malagasyprinus caeruleatus* (Lewis, 1905) comb. n. **12** 8^th^ sternite and tergite, ventral view **13** ditto, dorsal view **14** ditto, lateral view **15** 9^th^ and 10^th^ tergite, dorsal view **16** ditto, lateral view **17** aedeagus, dorsal view **18** apex of aedeagus, frontal view **19** aedeagus, lateral view **20** spiculum gastrale, ventral view **21** ditto, lateral view.

**Figures 22–24.**
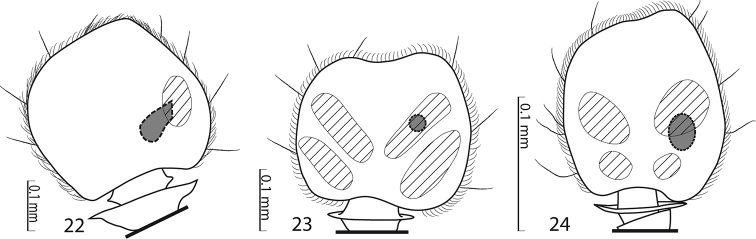
Sensory structures of the antenna of Saprininae. **22** *Malagasyprinus caeruleatus* (Lewis, 1905) comb. n., sensory structures of the antennal club, ventral view **23** *Saprinus* (s.str.) *semistriatus* (Scriba, 1790) sensory structures of the antennal club, ventral view **24** *Saprinus (Hemisaprinus) subvirescens* (Ménétriés, 1832) sensory structures of the antennal club, ventral view.

Antennal scape (Fig. 6) slightly thickened, with shallow sparse punctures and three short setae; antennal club (Fig. 5) round with slightly pointed tip, without visible articulation, entire surface with dense short sensillae intermingled with sparser longer erect sensillae; sensory structures of antennal club in form of a single oval sensory area situated on internal distal part of the antennal club with a corresponding stipe-shaped vesicle situated underneath (Fig. 22).

**Figure 25.**
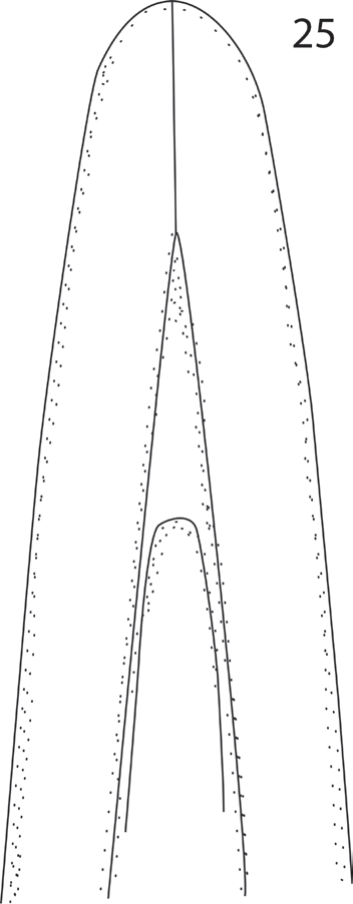
*Malagasyprinus perrieri* sp. n., apex of aedeagus, re-drawn from Dahlgren (1969).

**Figures 26–27.**
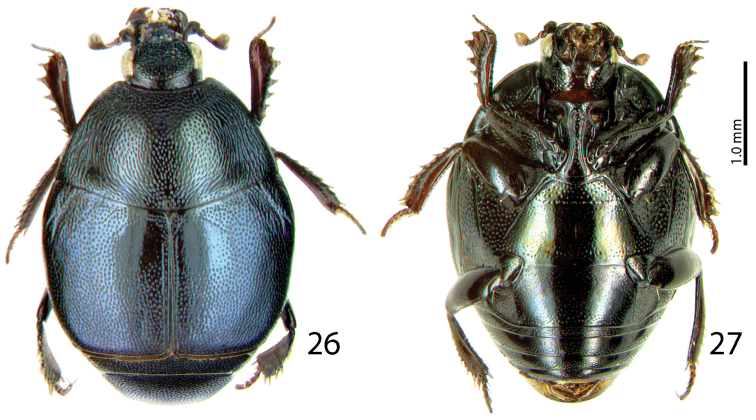
*Malagasyprinus perrieri* sp. n. **26** habitus, dorsal view **27** ditto, ventral view.

Mouthparts. Mandibles (Fig. 6) with rounded outer margin, laterally with deep dense punctures, moderately curved inwardly, mandibular apex pointed; sub-apical tooth on inner margin of left mandible large, almost perpendicular; labrum (Fig. 6) convex, imbricate; labral pits deep, each with two well-sclerotized long setae; terminal labial palpomere elongated, its width about half its length; mentum sub-trapezoid, anterior angles slightly produced; anterior margin (Fig. 7) medially with slight emargination surrounded with six long setae, lateral margins with row of sparse shorter ramose setae, several setae present also on disc of mentum; cardo of maxilla with few short setae; stipes triangular, with three short setae; terminal maxillary palpomere elongated, its width about one-third its length, approximately three times as long as penultimate.

Clypeus (Fig. 6) rugulose-lacunose, slightly depressed medially, sloping down laterally, faintly margined by prolonged frontal stria; frontal stria largely interrupted medially, for short distance prolonged onto clypeus, supraorbital stria well impressed, carinate; frontal disc (Fig. 6) depressed, coarsely and densely punctate, rugulose-lacunose; eyes strongly convex, well visible from above.

Pronotal sides moderately (Figs 1, 3) narrowing anteriorly, apical angles blunt, pronotal depressions absent; sides of pronotal disc with moderately deep longitudinal depression covered in deep longitudinal wrinkles, medially wrinkles disappear and become coarse and dense punctures; surface between longitudinal depression and pronotal margin slightly convex, punctate; marginal pronotal stria complete, laterally carinate and visible along its entire length from dorsal view; pronotal disc medially convex; pronotal hypomeron glabrous; scutellum small, but visible.

Elytral epipleura evenly punctate; marginal epipleural stria fine, complete; marginal elytral stria straight, well impressed and slightly carinate, continued as complete apical elytral stria. Humeral elytral stria weakly impressed on basal third, almost invisible under punctuation; inner subhumeral stria obliterated by coarse punctures; dorsal elytral striae (except for a tiny basal fragment of fourth dorsal elytral and complete sutural stria) completely erased by extremely coarse and dense elytral punctuation; fourth dorsal elytral stria present as short basal fragment connected with complete sutural elytral stria, which is apically connected with apical elytral stria; entire elytral disc (with exception of tiny punctate ‘mirror’ on fourth elytral interval) with extremely coarse and dense punctures, separated by less than half of their diameter; punctuation somewhat weakens before elytral apex.

Propygidium and pygidium (Fig. 10) densely and coarsely punctate, punctures separated by less than half of their diameter.

Anterior margin of prosternum (Fig. 8) almost straight; marginal prosternal stria present laterally; prosternal process on apical two-thirds concave, surface between carinal prosternal striae imbricate, laterally imbricate-punctate, punctures shallow; carinal prosternal striae well-impressed, carinae slightly divergent on prosternal apophysis, medially convergent and thence again divergent anteriorly, apically united under narrow loop; prosternal foveae large and deep; lateral prosternal striae carinate, sub-parallel, apically terminating near pre-apical foveae.

Anterior margin of mesoventrite almost straight, with median projection; discal marginal mesoventral stria well impressed, carinate, inwardly arcuate medially; disc of mesoventrite imbricate-punctate, punctures deep; meso-metaventral sutural stria undulate; intercoxal disc of metaventrite slightly depressed medially, with scattered round punctures of various sizes; lateral metaventral stria (Fig. 9) well impressed, carinate, almost straight, shortened; lateral disc of metaventrite (Fig. 9) slightly concave, with dense shallow punctures; metepisternum (Fig. 9) with even denser and coarser punctuation, punctures deeper than those of lateral disc of metaventrite; fused metepimeron with somewhat sparser punctures; metepisternal stria present only on fused metepimeron.

Intercoxal disc of the first abdominal sternite completely striate laterally; surface imbricate-punctate, punctures fine and sparse.

Protibia (Fig. 11) slightly dilated, outer margin with seven moderately large triangular teeth topped by short rounded denticle, diminishing in size in proximal direction; setae of outer row very short and sparse; protarsal groove deep, substrigulate; anterior protibial stria shortened apically; setae of median row shorter than those of outer row; two tarsal denticles present near tarsal insertion; protibial spur short, bent, inserted on apical margin of protibia; apical margin of protibia posteriorly with one tiny denticle; outer part of posterior surface imbricate, separated from imbricate median part of posterior surface by vague boundary and row of short sclerotized setae; posterior protibial stria complete, with a sparse row of tiny sclerotized setae becoming thicker apically; inner row of setae single, setae sparse and short.

Mesotibia slender, outer margin with two rows of sparsely spaced short denticles; setae of outer row regular, sparse, shorter than denticles; setae of median row regular, microscopic; posterior mesotibial stria complete; anterior surface of mesotibia imbricate; anterior mesotibial stria complete; mesotibial spur short; apical margin of mesotibia anteriorly with two short denticles; claws of apical tarsomere slightly bent, shorter than half its length; metatibia more slender and longer than mesotibia, in all aspects similar to it, but denticles on outer margin much sparser and claws of apical tarsomere slightly longer than half its length.

Male genitalia. Eighth sternite (Figs 12–13) fused medially, apically with short velum; apex fringed with two short setae; eighth tergite and eighth sternite fused laterally (Fig. 14). Ninth tergite (Figs 15–16) longitudinally fused medially, typical for the subfamily; spiculum gastrale (Figs 20–21) expanded on both ends; basal end with median emargination, not arcuate outwardly. Aedeagus (Figs 17–19) dilated medially; basal piece of aedeagus short, ratio of its length: length of parameres 1:5; parameres fused along their basal half; apex of aedeagus (Fig. 18) curiously split in two pointed halves curved inwardly; aedeagus strongly curved ventrad (Fig. 19).

#### Differential diagnosis.

From the highly similar *Malagasyprinus perrieri* sp. n., *Malagasyprinus caeruleatus* differs by smaller size; darker elytral cuticle (the elytra of *Malagasyprinus perrieri* are brown to dark brown whereas those of *Malagasyprinus caeruleatus* are pitch-black; compare Figs 1 and 26) shallower lateral longitudinal pronotal depression, and coarser elytral punctuation (in *Malagasyprinus perrieri* the elytral striae are more discernible whereas they are almost completely obliterated by punctuation in *Malagasyprinus caeruleatus*); furthermore, the elytral ‘mirror’ is often larger and less densely punctate in *Malagasyprinus perrieri* whereas it is tiny and often densely punctate in *Malagasyprinus caeruleatus*. The shape of carinal prosternal striae is likewise different between the two species: in *Malagasyprinus caeruleatus* they are strongly bi-sinuate, approximate medially and diverging, connected by a round loop, whereas in *Malagasyprinus perrieri* they are only slightly bi-sinuate, occasionally even sub-parallel (compare Figs 8 and 32) and furthermore, the prosternal process is medially deeply depressed in *Malagasyprinus caeruleatus*, whereas it is only slightly so with *Malagasyprinus perrieri*. However, the best marked differences are found among the aedeagi of the two species: in *Malagasyprinus caeruleatus* it is apically split in two inwardly curved halves resembling a snake’s tongue and in *Malagasyprinus perrieri* it is simply pointed apically and not split (compare Figs 18, 25 and 40). From *Malagasyprinus diana* sp. n., *Malagasyprinus caeruleatus* differs by its shallower lateral longitudinal depression of pronotum; furthermore, the deep longitudinal wrinkles occupy almost the entire pronotal disk in *Malagasyprinus diana*, whereas they are present mostly only in and around the longitudinal lateral pronotal depression with the median part of pronotum bearing simple punctures in *Malagasyprinus caeruleatus*. The elytral ‘mirror’ is proportionally larger in *Malagasyprinus diana* than in *Malagasyprinus caeruleatus* and it bears only sparse and fine punctures in *Malagasyprinus diana*, whereas it is coarsely and densely punctate in *Malagasyprinus caeruleatus* (compare Figs 1 and 44). More marked differences are found again between the shape of the carinal prosternal striae of the two taxa: those of *Malagasyprinus caeruleatus* are medially approximate and diverge anteriorly where there are connected by a round loop, whereas the carinal prosternal striae of *Malagasyprinus diana* are not approximate medially, slightly diverging on apical half (compare Figs 8 and 50). Prosternal process of *Malagasyprinus caeruleatus* is depressed on apical two-thirds whereas it is even in *Malagasyprinus diana*. Male aedeagi are likewise very different: the one of *Malagasyprinus diana* resembles that of *Malagasyprinus perrieri*, whereas the one of *Malagasyprinus caeruleatus* is unique with its split apex (compare Figs 18 and 60).

**Figures 28–34.**
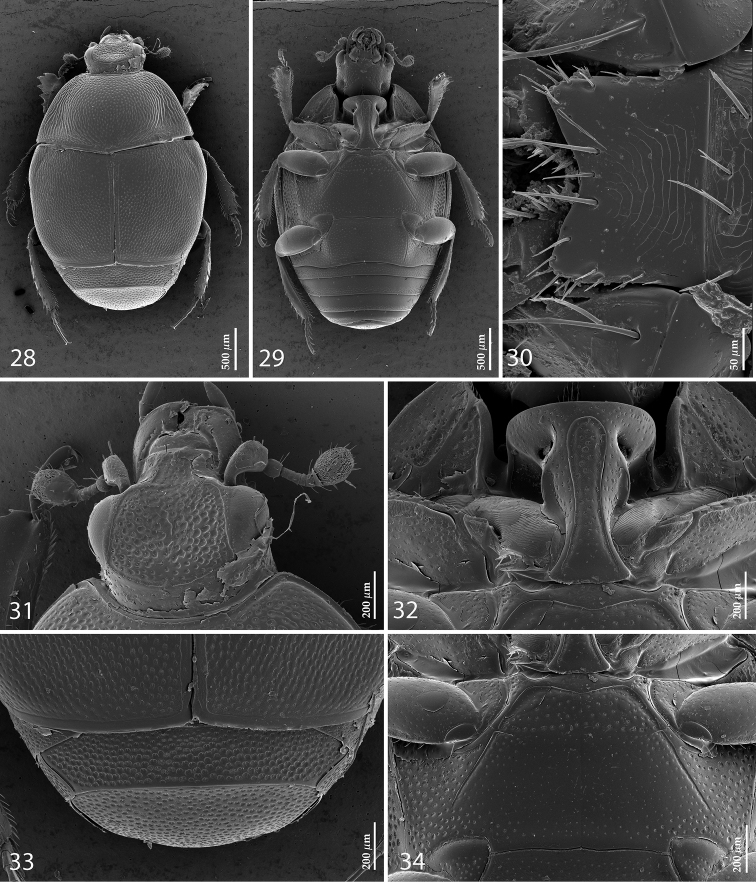
SEM micrographs *Malagasyprinus perrieri* sp. n. **28** habitus, dorsal view **29** ditto, ventral view **30** mentum, ventral view **31** head, dorsal view **32** prosternum **33** propygydium and pygidium **34** mesoventrite and metaventrite.

**Distribution.** Known exclusively from the south of Madagascar, regions of Antsimo-Andrefana, Androy and Anosy (Fig. 62).

**Biology.** Collected in dry forest by pitfall traps, as well as by sifting litter in spiny forest and/or thicket.

### Malagasyprinus perrieri sp. n.

http://zoobank.org/4F10C7F4-46B8-40D1-89DB-8CDD8A1CFD8B

http://species-id.net/wiki/Malagasyprinus_perrieri

Figs 25 –43

#### Type locality.

Madagascar.

#### Type material examined.

**MADAGASCAR:** Holotype, ♂, side-mounted on a triangular point, both antennal flagelli broken off, right fore- and mid legs missing, left mesotarsus missing, with male genitalia extracted and glued to the same mounting point as the specimen, with following labels: “♂” (printed), followed by light-green rectangular label, printed: “MUSEUM PARIS / MADAGASCAR / col. Perrier de la Bathie / 1906”; followed by hand-written label: “Saprinus / perrieri sp. n.” with a consecutive red label, printed: “*Malagasyprinus perrieri* / sp. n. Det. T. Lackner & / Y. Gomy 2013 HOLOTYPE” (MNHN). Paratypes: 31 exs., same data as holotype (MNHN); 1 ♂, with following labels: “♂” (written); “Madagascar N. Ouest / Ankarafantsika Ampisoro / 30.xi.1973 (written)”; “battage d’arbustes / (L. Linarès rec.) (written); “Collection / Y. Gomy (printed)”; “*Malagasyprinus perrieri* / sp. n. Det. T. Lackner & / Y. Gomy 2013 PARATYPE” (red label, printed)” (CYG); 2 ♀♀, with following labels: “♀” (written); “Madagascar N-Ouest / Ankarafantsika / Ampisoro 30.xi.1973 (printed label with black frame)”; “battage d’arbustes / (LLinarès rec.) (printed label with black frame)”; “Collection / Y. Gomy (printed)”; “*Malagasyprinus perrieri* / sp. n. Det. T. Lackner & / Y. Gomy 2013 PARATYPE” (red label, printed)” (CYG); 1 ♀, with the same labels as preceding, with an additional round, written label: “Photo / No 4 / 1^ére^ série”; 3 ♂♂, ibid (two of the male PT are sputter coated with gold) (CYG); 1 ♀, “MADAGASCAR: Mahajanga / Province, Parc National de / Baie de Baly, 12.4 km 337° / NNW Soalala, elev. 10m / 26–30 Nov. 2002 (printed)”; “16°00'36"S, 045°15'54"E / coll. Fischer, Grislwold et al. / California Acad. of Sciences / pitfall trap - in tropical dry / forest, coll. code: BLF6815 (printed)”; “CASENT / 8065522” (CAS); 1 ♀, same data, but “CASENT / 8065523” (CAS); 1 ♀, same data, but “CASENT / 8065521” (CAS); 1 ♀, same data, but “CASENT / 8065524” (CAS); 1 ♂, same data, but “CASENT / 8065520” (CYG); 1 ♂, same data, but “CASENT / 8065525” (TLAN); 1 ♀, same data, but “CASENT / 8065526” (TLAN); 1 ♀, same data, but “CASENT / 8065519” (TLAN); 1 ♀, same data, but “CASENT / 8065518” (CYG); 1 ♀, same data, but “CASENT / 8065517” (CYG).

#### Diagnosis.

**Figures 35–43.**
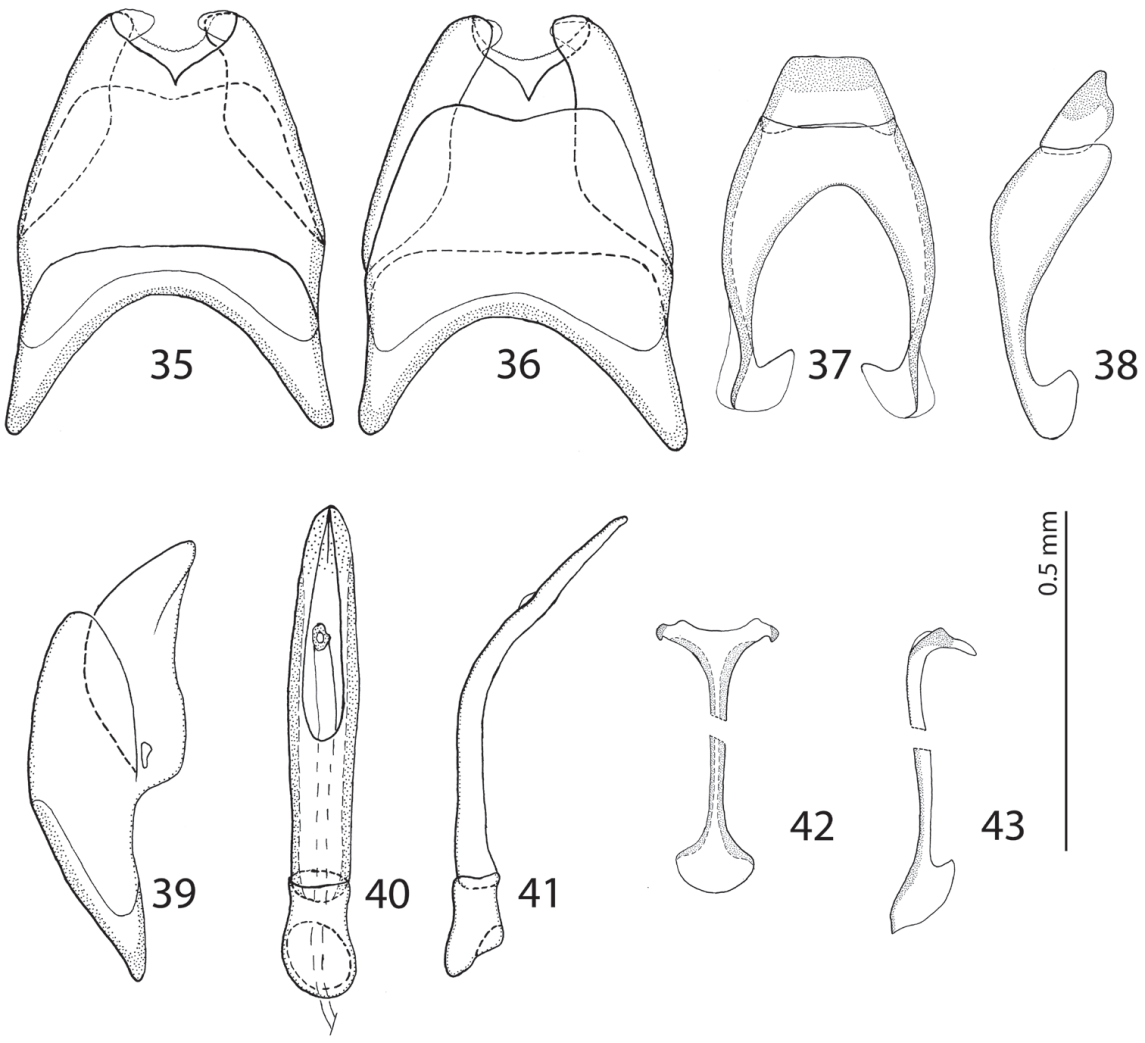
male terminalia *Malagasyprinus perrieri* sp. n. **35** 8^th^ sternite and tergite, ventral view **36** ditto, dorsal view **37** 9^th^ and 10^th^ tergite, dorsal view **38** ditto, lateral view **39** 8^th^ sternite and tergite, lateral view **40** aedeagus, dorsal view **41** ditto, lateral view **42** spiculum gastrale, ventral view **43** ditto, lateral view.

**Figures 44–45.**
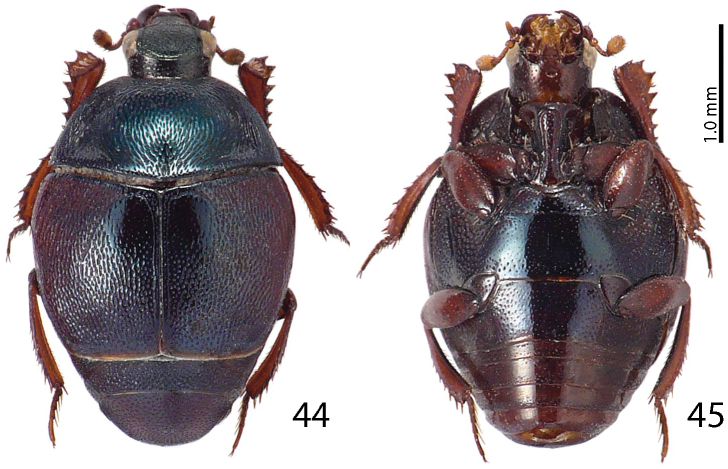
*Malagasyprinus diana* sp. n. **44** habitus, dorsal view **45** ditto, ventral view.

Body measurements: PEL: 2.20–2.60 mm; APW: 0.90–1.00 mm; PPW: 1.75–2.15 mm; EL: 1.25–1.50 mm; EW: 2.00–2.50 mm. Very similar to the preceding species, differing mainly by larger size; lighter color of legs and antennae (those of *Malagasyprinus perrieri* are brown to dark brown whereas those of *Malagasyprinus caeruleatus* are rufescent; compare Figs 1–2 and 26–27) deeper longitudinal pronotal depression, sparser elytral punctuation (in *Malagasyprinus perrieri* the elytral striae are more discernible whereas they are almost completely obliterated by punctuation in *Malagasyprinus caeruleatus*); furthermore, the elytral ‘mirror’ is often larger and less densely punctate in *Malagasyprinus perrieri* whereas it is tiny and often densely punctate in *Malagasyprinus caeruleatus* (compare Figs 1 and 26). The shape the of carinal prosternal striae is likewise different between the two species, see comments to the preceding species and compare Figs 8 and 32. Aedeagi of the two species are markedly different: that one of *Malagasyprinus caeruleatus* is apically split in two inwardly curved halves resembling a snake’s tongue and that one of *Malagasyprinus perrieri* is simply pointed apically and not split (compare Figs 18 and 40). From the following new species, *Malagasyprinus diana*, *Malagasyprinus perrieri* can be best distinguished by shallower longitudinal pronotal depression (the one of *Malagasyprinus diana* is the deepest among the three), the area of the pronotum covered by deep longitudinal wrinkles is the largest in *Malagasyprinus diana*, occupying almost the entire pronotal disk, whereas in *Malagasyprinus perrieri* it covers mostly the lateral pronotal depression and the surface around it; furthermore, the elytral ‘mirror’ is much smaller in *Malagasyprinus perrieri* than in *Malagasyprinus diana*, where it is proportionally the largest among the three taxa, and almost impunctate (compare Figs 26 and 44). The prosternal processes of the two species are likewise different: the carinal prosternal striae of *Malagasyprinus perrieri* are medially slightly approximate and rather narrowly separated, whereas those of *Malagasyprinus diana* are not approximate medially, widely separated and slightly divergent anteriorly (compare Figs 32 and 50). The prosternal process of *Malagasyprinus perrieri* is slightly depressed on its apical two-thirds, whereas that of *Malagasyprinus diana* is even. Male aedeagi (Figs 40 and 60) are similar between *Malagasyprinus diana* and *Malagasyprinus perrieri*, but the shape of 8^th^ sternite is different among species: in *Malagasyprinus perrieri* it is slightly more narrowing apically whereas in *Malagasyprinus diana* it is almost running parallel-sided (compare also Figs 35–36 and 53–54).

**Figures 46–52.**
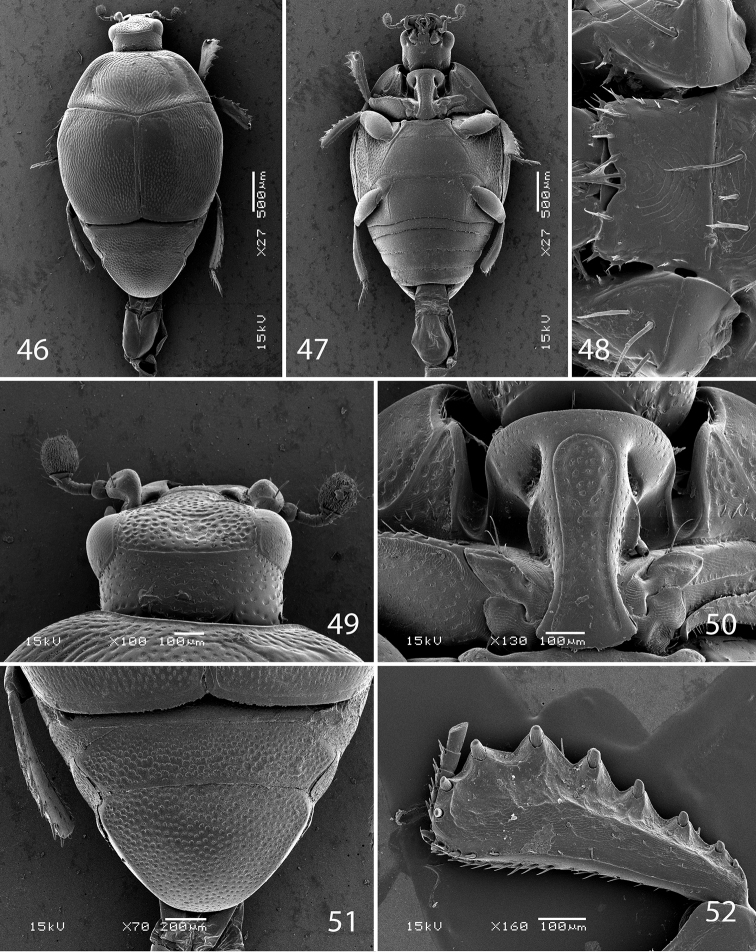
SEM micrographs *Malagasyprinus diana* sp. n. **46** habitus, dorsal view **47** ditto, ventral view **48** mentum, ventral view **49** head, dorsal view **50** prosternum **51** propygydium and pygidium **52** protibia, ventral view.

**Figures 53–61.**
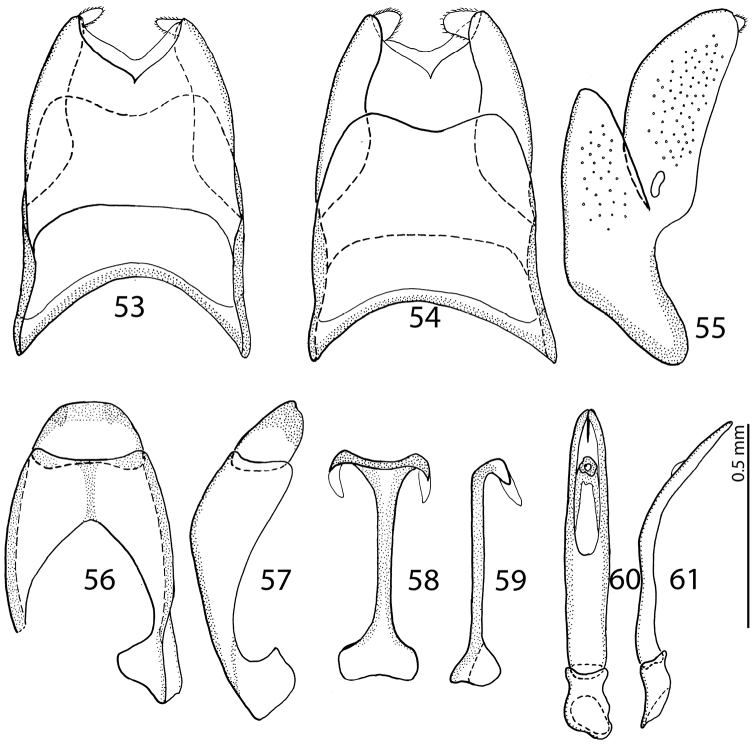
male terminalia *Malagasyprinus diana* sp. n. **53** 8^th^ sternite and tergite, ventral view **54** ditto, dorsal view **55** ditto, lateral view **56** 9^th^ sternite and tergite, dorsal view **57** ditto, lateral view **58** spiculum gastrale, ventral view **59** ditto, lateral view **60** aedeagus, dorsal view **61** ditto, lateral view.

#### Distribution.

**Figures 62.**
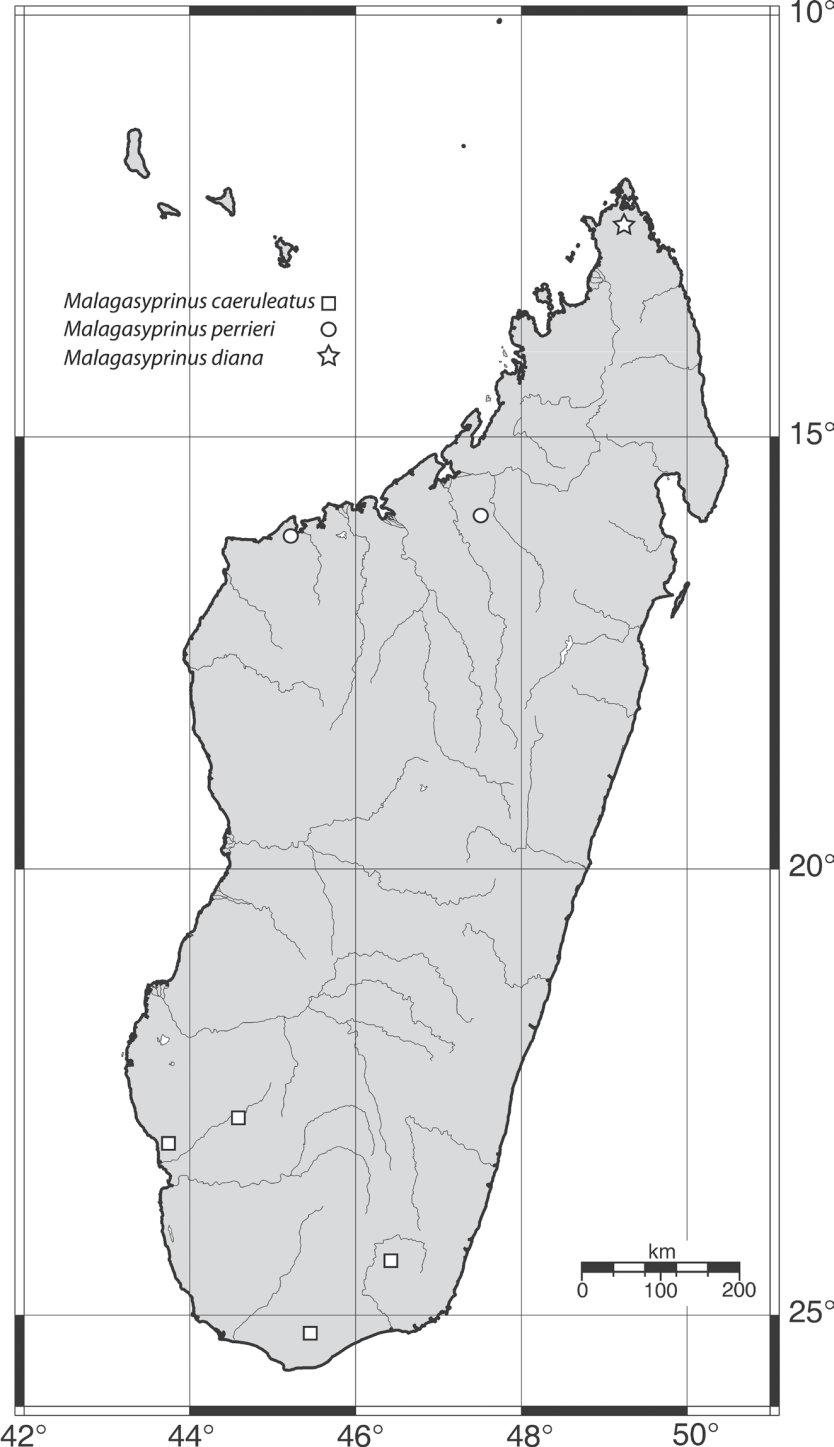
Map of distribution of *Malagasyprinus* gen. n.

*Malagasyprinus perrieri* is known from two localities, both situated in the region of Boeny, north-western Madagascar; see also Fig. 62 for the distribution of the three species).

#### Biology.

This species has been collected by beating the thickets as well as by pitfall trapping in tropical dry forest.

#### Remarks.

The specimens from Ankarafantsika (Ampisoro) slightly differ from those from national park of Baie de Baly in their punctuation of the ventral side of the body, but we regard these differences as variation between the two populations as the male genitalia are constant.

#### Etymology.

Patronymic, named in the honor of the first collector of this taxon, French botanist Henri Perrier de La Bâthie (1873–1958) well-known for his numerous studies of the Madagascar flora, who nonetheless collected also insects, currently deposited at MNHN.

### Malagasyprinus diana sp. n.

http://zoobank.org/93664EAE-B3E8-4FE8-9636-9D5E62F0E002

http://species-id.net/wiki/Malagasyprinus_diana

Figs 44 –61

#### Type locality.

Madagascar, Diana province, Forêt d’Orangea.

#### Type material examined.

**MADAGASCAR:** Holotype, glued on its side on a triangular mounting point, with male genitalia extracted, glued to the same mounting point as the specimen, with the following labels: “♂” (printed), followed by “MADAGASCAR: Province / d’Antsiranana Forêt / d’Orangea, 3.6 km 128° / SE Ramena Elev. 90m / 22–28 February 2001” (printed); followed by “12°15'32"S, 49°22'29"E” / coll. Fischer, Griswold et al. / California Acad. of Sciences / pitfall trap - littoral rainforest / collection code: BLF3127” (printed); followed by: “CASENT /8065747” (printed); followed by: “*Malagasyprinus diana* / sp. n. Det. T. Lackner & / Y. Gomy 2013 HOLOTYPE (red label, printed) (CAS). Paratypes (one of the male PT is sputter coated with gold): 1 ♂, same data, but “CASENT / 8065750” (CYG); 1 ♂, same data, but “CASENT / 80657501” (CAS). 1 ♂, same data, but “CASENT / 8065748” (TLAN). 1 ♀, same data, but “CASENT / 8065749” (CAS).

#### Diagnosis.

Body measurements: PEL: 2.20–2.30 mm; APW: 0.70–0.80 mm; PPW: 1.75–1.90 mm; EL: 1.40–1.50 mm; EW: 2.00–2.10 mm. Generally, this species has the deepest longitudinal pronotal depression and the largest area of the pronotum covered with deep longitudinal wrinkles. The elytral ‘mirror’ of *Malagasyprinus diana* is also the largest of the three species (compare Figs 1, 26 and 44 for the elytral sculpture of the three species). Furthermore, its carinal prosternal striae are rather widely separated, not approximate medially, only slightly diverging apically (compare Figs 8, 32 and 50 for the configuration of the prosternal striae among the three species) and the prosternal process is even, not depressed on anterior two-thirds. By the aedeagus (compare Figs 40 and 60) *Malagasyprinus diana* is most similar to *Malagasyprinus perrieri*, differing in the shape of 8^th^ sternite.

#### Distribution.

Known only from the northernmost tip of the island, region of Diana, northern Madagascar (Fig. 62).

#### Biology.

Collected in littoral rainforest by the method of pitfall trapping.

#### Etymology.

Patronymic, named after the region of Diana, where this species has been collected.

### Key to the species of *Malagasyprinus*

**Table:** 

1 (2)	Prosternal process even, carinal striae widely separated, not approximate medially, slightly divergent apically (Fig. 50)	*Malagasyprinus diana* sp. n. (extreme north of Madagascar)
2 (1)	Prosternal process depressed on anterior two-thirds, not even, carinal striae often approximate medially and thence divergent apically, occasionally united under round loop.
3 (4)	Carinal prosternal striae only slightly approximate medially, not substantially divergent apically (see Fig. 32), longitudinal pronotal depression rather deep, elytra brown to dark brown, aedeagus simply pointed, not split into two halves apically	*Malagasyprinus perrieri* sp. n. (west to north-west of Madagascar)
4 (3)	Carinal prosternal striae approximate medially, broadly divergent apically (see Fig. 8), united under round loop; longitudinal depression of the pronotum rather shallow, elytra pitch-black, aedeagus on its apex curiously split into two inwardly curved halves (see Fig. 18)	*Malagasyprinus caeruleatus* (Lewis, 1905) (southern Madagascar)

## Discussion

**Figures 63.**
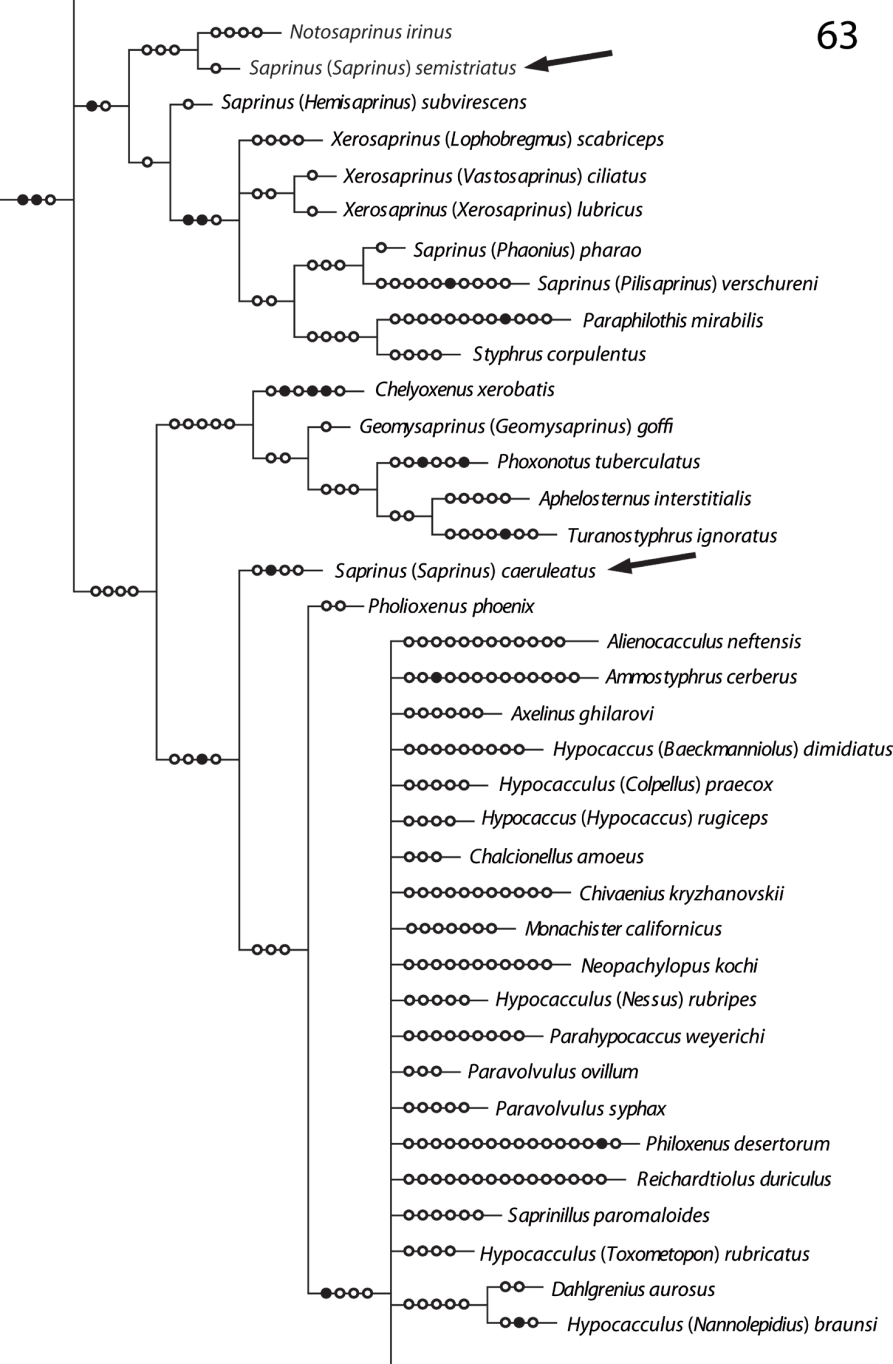
Position of *Saprinus semistriatus* and *Saprinus caeruleatus* on the phylogenetic tree (from Lackner, unpublished).

In the recently performed phylogenetic analysis focused on the resolving the relationships of the higher taxa of the Saprininae subfamily, the species *Saprinus* (s.str.) *caeruleatus* has been included alongside the type species of the genus, *Saprinus semistriatus* as there was a significant doubt that these two are actually congeneric. The reason for such doubt was mainly the conspicuously different structure of the sensory organs of the antennal club and the presence of large and deep prosternal foveae. The results of the yet unpublished analysis completely confirmed the initial suspicion as *Saprinus caeruleatus* has been placed distant from *Saprinus semistriatus* (Fig. 63), sister to a large and unresolved clade of genera that all share a unique synapomorphy of a single, stipe-shaped vesicle inside the antennal club, as well as several weaker synapomorphies, which are possibly homoplasies (Lackner, unpublished). The presence of the deep and large prosternal foveae exhibited by *Saprinus caeruleatus* is also completely unnatural among the members of *Saprinus*, with only the Palaearctic subgenus *Hemisaprinus* possessing them. As this character is mainly present among the more ‘derived’ members of the Saprininae subfamily, it is apomorphic. Another apomorphic character is the single sensory area with the corresponding stipe-shaped vesicle situated underneath on the internal-distal side of the antennal club, as the taxa that are placed near the root of the tree possess in most cases two or more vesicles inside their antennal club.

When Lewis (1905) famously remarked ‘I do not know of any species similar to this’ little did he know that his quip will provide a reason for a thorough study of the Malagasy specimens of ‘*Saprinus caeruleatus*’ which would yield another two, albeit very similar un-described species, and that his ‘*Saprinus caeruleatus*’ will rightly be awarded generic rank. It is highly likely that the three species of this newly erected genus share a single ancestor that reached Madagascar and subsequently speciated there. Based on the scant data available, we can hypothesize that the species *Malagasyprinus perrieri* and *Malagasyprinus diana* are closest relatives since their aedeagi are very similar, with *Malagasyprinus caeruleatus* as their common relative. However, the immediate ancestor of the three *Malagasyprinus* taxa is unknown, as we are unaware of a similar taxon either from Afrotropical region or from the Indian subcontinent.

## Supplementary Material

XML Treatment for Malagasyprinus

XML Treatment for Malagasyprinus caeruleatus

XML Treatment for Malagasyprinus perrieri

XML Treatment for Malagasyprinus diana
